# The Late Dr. Alden March

**Published:** 1869-06

**Authors:** 


The late Dr. Alden March.—The Albany papers contain extended obituaries of the late Dr. Alden March, who died on Thursday morning, 17th instant. He was born at Sutton, Mass., in 1795; graduated in the medical department of Brown University, Providence, R. I.; commenced the practice of his profession at Albany in 1820; was the founder of the Albany Medical College, which was organized in 1839, and of the Albany City Hospital. Among the last acts of his eventful life, he donated to these institutions, each $1,000. To the college he bequeathed his pathological museum, the most extensive and valuable in this country, with $1,000, the interest of which is to be perpetually employed for its care and preservation. To the hospital he had given the same amount, the interest to be expended for the purchase of surgical instruments for use in the hospital. As the *Argus* remarks, "His death will be regarded by the medical profession throughout the country as a loss to the cause of medical science. No surgeon in the United States, perhaps none in Europe, was held in higher estimation than Dr. March. Certainly no one in this country, in private practice alone, has performed so many bold and difficult and delicate surgical operations, and with such remarkable success. His reports of cases occurring in his practice, though few in comparison with the magnitude of his operations, have been received with the highest favor here, and widely republished in the medical magazines of Europe."

